# Health preferences and preventive care utilisation: How EQ-5D-5L health preferences may affect uptake

**DOI:** 10.1016/j.pmedr.2021.101514

**Published:** 2021-08-03

**Authors:** Dan Kelleher, Edel Doherty, Ciaran O'Neill

**Affiliations:** a Health Economics and Policy Analysis Centre, National University of Ireland, Galway, Ireland; b Centre for Public Health, Queen’s University Belfast, Ireland

**Keywords:** Ireland, EQ-5D-5L, Health preferences, Health-related behaviours, Health outcomes, Preventive care determinants

## Abstract

Despite the economic and health benefits of preventive care being well established, the uptake of many cost-effective preventive services remains lower than desired in many cases, especially among specific sub-populations. The value an individual places on health can influence their uptake of preventive care. One way to capture the value an individual places on health and future health status is to examine their health preferences. This study used a novel use of EQ-5D-5L health preferences to determine if health preferences are associated with the uptake of a range of preventive care services, including a cancer screening, blood pressure check, cholesterol check, blood test and urine test. We collected EQ-5D-5L composite time trade-off data in 2018/2019 on 242 respondents residing in Ireland. We estimated an initial tobit model to predict an individual’s health preference to capture health preferences as a regressor. We then estimated a bivariate probit model to examine the uptake of each preventive service and GP use. Each model controlled for health preferences, education, sex, type of health coverage, self-reported health, employment status, age and marital status. Health preferences are a significant determinant of all five preventive services while controlling for other covariates. The results shows that the higher an individual values good health, the more likely they are to avail of preventive care. Health preferences can be noted as a potential determinant of preventive care use that could guide policy responses seeking to increase demand-side factors for preventive care uptake.

## 1 Introduction

An extensive body of literature exists that examines socio-demographic factors in explaining the uptake of preventive care services (Burns et al., 2012, Hsia et al., 2000, Walsh et al., 2016). Despite the economic and health benefits of preventive care being well established, the uptake of many cost-effective preventive services remains lower than desired in many cases, especially among specific sub-populations (Sabbath et al., 2018, Meier, 2000, Nolan et al., 2019). For policymakers to address this issue, they must clearly understand the factors that underpin preventive care uptake. Especially in countries where access to preventive care is free at the point of use and where readily identifiable barriers to care may not exist.

Within the broader literature, studies have found that lower uptake of preventive care is associated with lower levels of education, health literacy and household income, as well as a lack of usual source of healthcare provider, health insurance coverage and migrant status (Carney and O’Neill, 2018, Sambamoorthi and McAlpine, 2003, Mandrik et al., 2021, Norredam et al., 2010). Various other factors have also been shown to influence an individual’s use of preventive care. Carrieri and Bilger (2013) note that health beliefs, which are the values and attitudes that an individual places on health, can influence their use of preventive care. Walsh and McPhee (1992) also highlight that health value orientation (the value an individual places on health) is a key component of an individual’s preference for preventive care, as individuals who place a high value on health undertake more preventive care. Shi et al. (2004) highlight the importance of health values and note that individuals who place a high value on health have greater healthcare-seeking practices than those who do not. One way to capture the value an individual places on health and future health status is to examine their health preferences.

Health preferences can be captured by an individual placing utility valuations on measures of health that combine quality of life and time, such as using the EQ-5D-5L health descriptive system (Oppe et al., 2016). One such way to capture health preferences is to examine an individual’s valuation of the (55555) health state, as it the most severe health state an individual is asked to value and the only health state all respondents value in EQ-5D-5L valuation studies (Hobbins et al., 2018, Devlin et al., 2018, Ludwig et al., 2018). For example, Barry et al. (2018) used the (55555) health state and other severe health states to capture individual health preferences in examining how health preferences may be related to an individual’s religious views. Health preferences are noted to be indicative of various individual health-related behaviours, such as healthcare use, health-promoting behaviours, willingness to engage in unhealthy behaviours and how one determines the perceived value of an investment in health capital (Kelleher et al., 2020, Hobbins et al., 2020).

Having knowledge of individual health preferences may provide insight into further determinants of preventive care use since health preferences can capture the value an individual places on health and future health status. Indeed, health preferences may have been an unobserved variable in previous studies examining the determinants of preventive care uptake. It is, therefore, worthwhile to determine if health preferences are associated with an individual’s preventive care use. That is, in particular circumstances, health preferences may not influence an individual’s access to preventive care, but they may influence an individual’s desire to avail of such care. Without understanding how health preferences affect an individual’s uptake of preventive care, policies that seek to increase uptake may underestimate this potential factor's role in influencing uptake of such services.

This paper presents an exploration of how variations in health preferences may relate to preventive care uptake. This paper uses a novel use of health preferences generated using EQ-5D-5L technology to capture and examine how an individual’s health preferences may be associated with their uptake of preventive care. Understanding how health preferences may relate to preventive care uptake adds to the literature on the determinants of preventive care use. This area of research has not been examined in the literature to the best of our knowledge previously. Specifically, this paper examines if an individual’s health preferences are associated with their uptake of various preventive services, including a cancer screening, blood pressure check, cholesterol check, blood test and urine test in Ireland. The results of this study may be of considerable interest to policymakers charged with ensuring preventive care uptake is sufficient within Ireland and elsewhere.

## 2 Methods

### 2.1 Health preferences

The EQ-5D-5L generic health measure provides a descriptive framework for the characterisation of health status. Numerous valuation studies that are grounded in validated preference elicitation technology have been undertaken that measure individual preferences for health states using the EQ-5D-5L health descriptive system (Devlin et al., 2018, Ludwig et al., 2018, Doherty et al., 2021). The EQ-5D-5L measures health-related quality of life (HRQoL) across five dimensions, namely: mobility, self-care, usual activities, pain/discomfort and anxiety/depression. Each dimension is categorised by one of five levels of severity: no problems, slight problems, moderate problems, severe problems and extreme problems (Hobbins et al., 2018).

To capture health preferences, data are elicited through composite time trade-off (cTTO) exercises by placing utility valuations on EQ-5D-5L health states (Oppe et al., 2014). Using a series of cTTO exercises, it is possible to determine the length of life an individual is willing to forego to live in a better health state (i.e. with full health) and avoid living in a health state with compromised health (Oppe et al., 2016). EQ-5D-5L health state utility values are bound between −1 (a state considered to be worse than dead) and 1 (full health), with states anchored at 0 for dead. In the cTTO exercises, an individual is asked to compare and value two hypothetical health states to determine which is better using an iterative procedure to vary time spent in the states. Health state A is always described as ‘full health’ and is compared to a health state B, which is always described as a compromised health state using one level for each of the five EQ-5D-5L health dimensions, as noted above. Each health state is denoted by using a series of five numbers to represent the respective severity levels of each health dimension. For example, health state (23245) represents a health state with slight problems in mobility, moderate problems in self-care, slight problems in usual activities, severe pain/discomfort and extreme anxiety/depression. The use of cTTO data to examine EQ-5D-5L health preferences is common in the literature (Kelleher et al., 2020, Barry et al., 2018, Hobbins et al., 2020, Pickard et al., 2013, Shaw et al., 2007, Sayah et al., 2016).

More detail on cTTO health states and how the utility values are derived can be found in supplementary material and in the paper by Oppe et al. (2016).

## 3 Data set

We collected cTTO data from 242 individuals residing in Ireland, with each individual valuing six practice states plus 11 real cTTO states (from one out of a possible three blocks of cTTO states). All interviews were collected using the EuroQol Research Foundation's EuroQol-Portable Valuation Technology (EQ-PVT) and protocol (Welie et al., 2020, Stolk et al., 2019). An additional survey was used to capture an individual’s healthcare use after completing the cTTO exercises. Respondents were asked in this survey whether they had any cancer screening, blood pressure checks or cholesterol checks in the previous 24 months. Within this survey, respondents were also asked if they had any blood tests, urine tests and a general practitioner (GP) visit during the previous 12 months. We adapted published self-reported healthcare use surveys, which had used the above timelines when enquiring about the use of those particular services (Lairson et al., 2009, Ritter et al., 2001, Reijneveld, 2000).

A range of socio-demographic questions was asked and recorded in the EQ-PVT. Data were collected from June 2018 to September 2019. The full sampling strategy and survey design can be seen in the supplementary material.

### 3.1 Statistical analysis

In Ireland, general practitioners (GPs) are primary caregivers and gatekeepers to other diagnostic and specialist services (Heavey, 2019). As such, it is then appropriate to use to model preventive care utilisation and GP visits simultaneously (McGregor et al., 2006, Carrieri and Bilger, 2013). A bivariate probit model was used to examine the uptake of each preventive service (a cancer screening, blood pressure check, cholesterol check, blood test and urine test) and GP use. This modelling approach accounts for possible correlation among unobserved components of the two regression equations, thereby capturing the correlation between unobserved variables that influence an individual’s uptake of each preventive service and their GP use (Barry et al., 2018, Bíró, 2013, Kotwal et al., 2016). Uptake for each preventive service and GP use was specified as a function of health preferences and a range of socio-demographic characteristics, including education, sex, type of health coverage, self-reported health (visual analogue scale (VAS)), employment status, age and marital status.

## 4 Modelling health preferences

To capture health preferences as a regressor, we estimated an initial tobit model to predict an individual’s health state utility valuation for health state (55555) (this is the only health state that all individuals valued using the cTTO method). A tobit model was used to account for the censored nature of the data, which censors the data at −1. Health state (55555) is the most severe health state an individual is asked to value, reflecting a state with the worst levels across all five dimensions of the EQ-5D-5L. The initial tobit model controlled for; age, sex, marital status and urban household location as these covariates are noted as significant predictors of cTTO health state utility valuations (Sayah et al., 2016), and the main effects which are the 20 dummy indicators for each of the levels of the EQ-5D-5L dimensions recorded as = 1 if a given level of a given dimension is present and = 0 if not. The base category representing the best scenario in each dimension (i.e., level 1 = no problems) is the omitted category in the model. The estimated value for each individual’s utility valuation for health state (55555) was then included as a regressor to capture a respondent’s health preferences in the bivariate probit models on using preventive care and GP visits alongside a respondent’s socio-demographic characteristics.

It is important to that note that we could include the predicted utility value for any health state into the regressions examining each preventive service, and get the same regression coefficients. The predicted health state utility value is a liner function of the health state dimensions with the difference being the same across the utility function. The utility function is a linear index of the health state with the differences predicted for each individual reflecting differences in the observed socio-demographic characteristics included in the original tobit model, which are an individual’s age, gender, marital status and location of their household, which are noted as significant predictors of cTTO utility values (Sayah et al., 2016), as already noted.

When we predict the health state utility value for any health state, and include it as a regressor we would get the same results as those produced later in this paper. This finding is to be expected as the predictions for each individual differ by the coefficients on the health states so they are colinear.

(1) $$Pred=X_{1}\beta_{1}+X_{2}\beta_{2+}X_{3}\beta_{3}+X_{4}\beta_{4}+X_{5}\beta_{5}$$

(2) $$P_{33333}=3\beta_{1}+3\beta_{2}+3\beta_{3}+3\beta_{4}+3\beta_{5}$$

(3) $$P_{55555}=5\beta_{1}+5\beta_{2}+5\beta_{3}+5\beta_{4}+5\beta_{5}$$

Since the betas are constant, if we include any predicted health state utility value we would get the same regression coefficients as those in the five preventive care use models which control for health preferences presented later in this paper.

Ethical approval for the study was granted by NUI Galway's Research Ethics Committee (application number 18-Mar-13). All of the analysis was carried out using STATA-16.

## 5 Results

The descriptive statistics of the sample and how each covariate was coded is displayed in Table 1, Table 2. The results from the initial tobit model are displayed in Table 1A of the supplementary material. For the ease of interpreting the results, the estimated health state utility value for health state (55555) for all individuals was rescaled into a disutility value instead of a utility value, which is common when using EQ-5D-5L cTTO data (Kelleher et al., 2020, Kharroubi et al., 2010, Hobbins et al., 2018)). The disutility scale is now bounding the health state utility values between 2 (worse than death) and 0 (full health). The mean estimated utility value for health state (55555) was 1.8 (SD: 0.06).

**Table 1:** Sample descriptive statistics.

Variable	Mean (SD) for continuous variable Or % for categorical variables
Health preference (continuous)	1.8 (0.06)
Third level education (1 = yes)	71% (0.45)
Sex (1 = male)	39% (0.49)
Health coverage: (base = no coverage) (1 = yes)	33% (0.47)
Government-funded medical card (2 = yes)	17% (0.38)
Private health insurance (3 = yes)	50% (0.5)
VAS (continuous: 100 = full health; 0 = worst health possible))	84.5 (10.98)
Employed (1 = yes)	65% (0.48)
Average age (1 = ≤ average age of sample (36 years))	55% (0.5)
Married (1 = yes)	48% (0.5)
Number of observations	242

**Table 2:** Sample descriptive statistics for the preventive care services.

Variable	Mean (SD) for continuous variable Or % for categorical variables	Mean Age (SD)	Mean Sex (SD)	Mean VAS (SD)
GP use (1 = yes)	80% (0.4)	36 (12.38)	35% (0.48)	84 (11.28)
Cancer screening (1 = yes)	42% (0.49)	40 (12.59)	20% (0.4)	84 (12.08)
Blood pressure test (1 = yes)	67% (0.47)	38 (12.5)	35% (0.48)	83 (11.41)
Cholesterol check (1 = yes)	46% (0.5)	41 (12.17)	34% (0.48)	83 (12.17)
Blood test (1 = yes)	62% (0.49)	38 (12.43)	33% (0.47)	83 (11.67)
Urine test (1 = yes)	32% (0.47)	38 (13.67)	31% (0.46)	82 (12.19)
Number of observations	242			

Mean Age, Sex and VAS are calculated for respondents who availed of each preventive service.

Table 3 presents the rho statistic for each of the five bivariate probit models. The different preventive services and GP use are the dependent variables across the five bivariate probit models. The results show that rho, which is the correlation coefficient for the two error terms from each model, is highly significant and positive in all models, signifying a strong positive correlation between the two regressions of each model. This positive correlation suggests that where we over (under) predict the use of GP services, we over (under) predict the use of the preventive service concerned.

**Table 3:** Bivariate probit rho statistic for each model.

Preventive service & GP use	Log-likelihood	Rho	p-value
(1) Cancer screening & GP use	−2492.7543	0.281	0.0481**
(2) Blood pressure & GP use	−2520.7852	0.518	0.0002***
(3) Cholesterol & GP use	−2483.9063	0.332	0.0179**
(4) Blood test & GP use	−2537.9849	0.408	0.0016***
(5) Urine test & GP use	−2622.1755	0.380	0.0066***

***p < 0.01 **p < 0.05 *p < 0.1

In Table 4, the average marginal effects for the five models examining each preventive service are presented. An individual’s health preferences are a significant determinant of preventive service use, with the results demonstrating that the greater disutility (lower utility) valuation an individual places on compromised health, the more likely they are to have had a cancer screening (p < 0.01), blood pressure check (p < 0.01), a cholesterol check (p < 0.05), blood test (p < 0.05) or urine test (p < 0.1). The magnitude of the marginal effects suggests that an individual’s health preferences have the greatest bearing on the probability of having had a cancer screening, followed by a cholesterol check, a blood test, a blood pressure check, and a urine test. The results from the other remaining independent variables follow intuition, for example, men are less likely to use both preventive care and primary care compared to women; those with private health insurance are more likely to use cancer screening services compared to those with no health coverage; the better an individual self-rates their health status the less likely they are to have availed of a blood pressure check or a blood test. Variations in health preferences are not seen to be significantly related to the use of GP services, and these results are displayed in Table 2A of the supplementary material.

**Table 4:** Bivariate probit average marginal effects for each preventive service.

	(1) Cancer screening		(2) Blood pressure		(3) Cholesterol check		(4) Blood test		(5) Urine test	
Variable	Coefficient	(SE)	Coefficient	(SE)	Coefficient	(SE)	Coefficient	(SE)	Coefficient	(SE)
Health preference	2.444***	0.596	1.702***	0.729	2.138***	0.606	1.892***	0.659	1.093*	0.618
Third level education	0.117*	0.065	−0.042	0.063	−0.026	0.064	−0.060	0.065	0.007	0.068
Sex	−0.366***	0.058	−0.137**	0.066	−0.088	0.063	−0.150***	0.064	−0.113*	0.064
§Health coverage	(base: no coverage)									
§Medical card	0.181**	0.086	0.137	0.088	0.102	0.090	0.207***	0.083	0.059	0.097
§Private insurance	0.132**	0.064	0.150**	0.068	0.141**	0.064	0.034	0.068	−0.004	0.065
VAS	0.001	0.002	−0.005**	0.003	−0.004	0.003	−0.007***	0.003	−0.005**	0.003
Employed	0.052	0.059	0.105*	0.063	0.109*	0.062	0.111*	0.065	0.042	0.062
Age	0.246***	0.063	0.053	0.086	0.063	0.077	0.187***	0.075	0.143*	0.081
Married	0.251***	0.071	0.026	0.074	0.339***	0.076	0.269***	0.075	0.156**	0.074
Number of observations	242		242		242		242		242	

***p < 0.01 **p < 0.05 *p < 0.1

Dependent variable = whether or not a respondent had use of each preventive service.

SE = standard error.

§base/reference category = no health coverage.

More descriptive statistics are displayed in Tables 3A, 4A, 5A, 6A and 7A of the supplementary material, but due to brevity are not discussed here.

## 6 Discussion

Our study provides a novel use of EQ-5D-5L health preferences to examine how an individual’s health preferences are associated with the uptake of preventive care. Previous studies have examined a multitude of determinants of preventive care uptake. However, to the best of our knowledge, our study is the first to explicitly examine how an individual’s health preferences may be associated with the uptake of a range of preventive care services.

The results from the study denote that health preferences are associated with an individual’s uptake of a cancer screening, blood pressure check, cholesterol check, blood test and urine test while controlling for other covariates. The interpretation of the results for all of the above listed preventive services is clear and intuitive. The greater disutility (lower utility) valuation an individual places on compromised health, the more likely they are to have had uptake of all preventive services. Such that, when an individual is faced with placing a value on a compromised health state, the worse an individual perceives compromised health to be, and how adversely it will affect their future health status the more likely they are to have use of preventive care.

For the ease of interpreting the results, it is useful to compare the marginal effects associated with health preferences and each preventive service to the marginal effects associated with the other covariates. For example, in Table 4, we can see that marital status is a strong and positive predictor of preventive care uptake in all models, consistent with the literature (Kotwal et al., 2016). Similarly, there is a strong and positive association between health preferences and each preventive care service as the greater disutility valuation an individual places on compromised health, the more likely they are to have had uptake of each preventive services. The results of this study may resonate as a note of caution in assuming that a cancer screening has a higher marginal effect than that associated, for example, with a urine test, but this still may be informative. It may, for example, be indicative of a difference in perception regarding the value of information from a cancer screening compared to, say, a urine test. The effect of health preferences on the likelihood of preventive care use might be magnified, perhaps where the threat to health is seen as greater or more immediate.

Health preferences can be noted to underpin health-related behaviours and healthcare use, as individuals who place a higher value on health states are noted to have different health-related behaviours and healthcare use than those who do not (Hobbins et al., 2020). By determining the value an individual places on health states, such as using EQ-5D-5L health state utility valuations, it is possible to determine the relative value an individual places on health and future health status. The higher an individual values health is shown to influence their use of healthcare, especially preventive care, as mentioned above. This study shows that the higher an individual values good health, the more likely they are to have availed of preventive care. This finding is consistent with those of the previously cited papers (Carrieri and Bilger, 2013, Walsh and McPhee, 1992, Shi et al., 2004), which all note the better an individual values health, the greater uptake of preventive care they have.

We examined preventive care uptake and GP use jointly and the importance of doing so is evident from the analysis and the significance of rho in each of the five bivariate probit models. Rho is positive in each model, and this would suggest that where we over (under) predict the use of GP services, we over (under) predict the uptake of preventive care. These findings can be interpreted regarding unobserved variables omitted from the analysis. For example, rate of time preference (future discount rate) was an omitted variable that is noted to determine healthcare use, especially preventive care. Individuals who have a low rate of time preference have greater uptake of cancer screenings, cholesterol testing and vaccinations (Bradford, 2010).

We note some limitations to the current study. We were constrained in sample size, and the use of preventive care in our healthcare use survey is a subjective accounting from each individual, which may not be wholly accurate. Some potentially relevant information was missing from our data (such as time preference), which may have influenced our modelling results. Our ability to measure other variables was perhaps somewhat crude; we were not, for example, able to capture an individual’s health history or that of their family. Further research with a larger sample size may still be required in this area to examine further how health preferences and preventive care use are related. This paper has provided the initial building block to examining a potential new factor that can underpin an individual’s uptake of preventive care.

EQ-5D-5L national valuation studies are an extremely rich source of data, which are pivotal in guiding healthcare resource allocation decisions (Pickard et al., 2019, Yang et al., 2019). Moreover, these valuation studies can provide a wealth of information on individual health-related behaviours that can be used to inform an array of health policies. For example, such information could inform migrant health policy seeking to increase the uptake of preventive care by migrants. Migrants have lower use of preventive care when compared to host populations, and these differences are noted to be potentially underpinned by differences in health preferences (Kelleher et al., 2020).

This paper has added to the literature by examining health preferences and the use of preventive care. An extensive literature exists examining the many determinants of preventive care uptake, such as health insurance coverage and migrant status and the intended policy responses to increase uptake with more detailed above. Various policies will utilise the information on the determinants of preventive care use in a bid to increase the required uptake of these services. For example, expanding entitlements for those with limited cover health insurance, making preventive care free at the point of use or highly subsidised and increasing health information campaigns to promote and encourage the use of preventive care. While such policies may remove barriers to service use, they will not, and should not, ensure service uptake where health preferences may underpin differential uptake. Our analysis may provide a mechanism by which policymakers can assess whether disparities in the use of preventive care are grounded in legitimate differences in health preferences or what might be considered to be illegitimate differences in access. The consideration of health preferences is certainly worthy of consideration in the framing of policy.

## 7 Conclusion

This paper has shown by using a novel use of EQ-5D-5L health preferences that health preferences may determine an individual’s use of preventive care. These findings highlight that health preferences can be noted as a potential determinant of preventive care use, and they add to the literature on the determinants of preventive care uptake. The use of EQ-5D-5L health preferences is instrumental in health policy by guiding the allocation of healthcare resources; the same health preference could also prove pivotal in examining individual health-related behaviours to inform various health policies.

## 8 Funding

This study was funded by the Hardiman Research Scholarship 2017 awarded by NUI Galway to Dan Kelleher.

## 9 Authorship and Originality

The corresponding author warrants that all aforementioned authors fulfill the criteria of authorship as defined by the International Committee of Medical Journal Editors (ICMJE). The corresponding author further warrants that the work described in this manuscript has not been published before and is not (nor will be) under consideration elsewhere while under review in *Preventive Medicine*; that all authors approved the present submitted version and their institutions have no objections to the manuscript’s contents.

## 10 Ethical compliance

The corresponding author warrants that if the manuscript describes research on human subjects the necessary ethical approval (or exemption) has been obtained and is on file with the authors’ institutions. For empirical research papers, add a statement of ethical compliance or exemption to the Methods section.

## CRediT authorship contribution statement

**Dan Kelleher:** Conceptualization, Data curation, Formal analysis, Funding acquisition, Investigation, Methodology, Project administration, Software, Supervision, Validation, Writing - original draft, Writing - review & editing. **Edel Doherty:** Supervision, Conceptualization, Writing - review & editing. **Ciaran O’Neill:** Supervision, Conceptualization, Writing - review & editing.

## Declaration of Competing Interest

The authors declare that they have no known competing financial interests or personal relationships that could have appeared to influence the work reported in this paper.
