# Supplementary material for: Health preferences and preventive care utilisation: How EQ-5D-5L health preferences may affect uptake
Source: Prev Med Rep. 2021 Aug 3;24:101514. doi: 10.1016/j.pmedr.2021.101514 (PMC8358689; doi:10.1016/j.pmedr.2021.101514)
Supplement: Supplementary Data 1 [file mmc1.docx]

**Supplementary Material**

**Extended survey details**

***Sampling Strategy***

Health preferences were captured through health state utility valuations and elicited through composite time trade-off (cTTO) exercises (Oppe et al., 2016). Each respondent valued six practice states plus one block of 11 cTTO states all in English. There are 11 observations per respondent. Data were collected from June 2018 to September 2019 with the EuroQol Research Foundation advising on the study sample size and providing the orthogonal design of health states for valuation. All interviews were collected using the EuroQol Research Foundation's EuroQol-Portable Valuation Technology (EQ-PVT). The EQ-PVT is PowerPoint replica of the EuroQol-Valuation Technology (EQ-VT), which has been used in previous studies ((Welie et al., 2019); (Stolk et al., 2019)). Ethical approval for the study was granted by NUI Galway's Research Ethics Committee (application number 18-Mar-13).

***Experimental Design***

We used a similar orthogonal design as used by Yang et al. (2019) who note an orthogonal design was generated by assigning dimensions and levels to a pre-existing orthogonal array, and for the EQ-5D-5L, an orthogonal main-effect design mathematically contains a minimum of 25 health states. The authors further detail, orthogonal designs hold a theoretical advantage as the absence of correlations between dimensions offers a strong basis for decomposing the observed values to the underlying dimension of severity levels with each level occurring equally often within each dimension. The orthogonal design entails of 25 health states including the 55555 *'pits'* state plus five mild health states to compensate for an under-representation of mild states, for a total of 30 EQ-5D-5L health states to be valued.

***Blocking***

The 30 health states were divided into three blocks (A, B and C) using the blocking algorithm included in the "*AlgDesign*" package in R. The blocking algorithm divides the states over the blocks to ensure that within block variance is maximised so that the full utility range is achieved. All blocks contained an equal number of health states and are perfectly balanced in terms of the utility range and misery index. The misery index is a proxy measure of the severity of the health state and is calculated by adding together the levels of each health state (for example, state 33252: 3 + 3 + 2 +5 + 2 = 15).

***Interviewers and Respondents***

We collected cTTO state valuations for the three blocks of health states for adults aged 18 years and over living in Ireland. Five interviewers and one study coordinator collected interviews. Respondents were recruited into the study through a Facebook study page, emailing the study coordinator or being contacted through friends and family using snowball sampling. All respondents received a €10 shopping voucher for participating in the survey. All interviewers received the standard three days of training before conducting real interviews. The training consisted of, learning about QALYs, the EQ-5D questionnaire, the use of the EQ-PVT software, and carrying out practice surveys. Interviews were conducted in the respondents home or another prearranged location. Respondents provided written consent to participate in the study, followed by socio-demographic questions, including their self-report health using the EQ-5D-5L descriptive system and the visual analogue scale (VAS). Finally, respondents completed 17 cTTO tasks including six practice states made up of three wheelchair example states (wheelchair, a situation much worse than a wheelchair, and a situation much better than a wheelchair) and three example EQ-5D-5L states (21121, 15411, 35554). After the six practice states were complete, and the respondent was comfortable with the cTTO tasks, they then proceeded to answer the 11 real cTTO tasks. A feedback module was provided to respondents. All interviewers adhered to the EuroQol Protocol and guidelines for quality control checks (Stolk et al., 2019). Quality control reports were sent to EuroQol after every 50 completed interviews to assess the performance of each interviewer and the quality of the data.

***cTTO Data***

For this study, a cTTO exercise was used to elicit health preferences. The lowest achievable cTTO utility value is −1 with this value representing a health state so severe that a respondent has traded off all available time. However, it is important to note that while the elicited utility value is bounded at −1 due to the elicitation method, it is theoretically possible that the disutility associated with a given health state may be greater still. It is plausible that a respondent could potentially assign a value lower than the bounded −1 to a given health state if the elicitation method allowed. It might be possible that a health state could be considered so much worse than death, that the respondent would be willing to trade off any amount of time to avoid this, corresponding to a utility value of minus infinity. Thus, the true utility score runs from minus infinity to plus one. The highest achievable cTTO utility value is 1 representing a health state so inconsequential that a respondent has not traded off any available time. See the paper by Oppe et al. (2016) for further detail of cTTO health states.

**Calculating a health state utility value for a better than dead state**

An individual is asked to decide if it is better to live a shorter life in health state A with full health or a longer life in a health state B with compromised health. In the iterative process, the amount of time in health state A (full health) is altered on a scale of one to ten years, while the amount of time in a health state B with compromised health is fixed at ten years until a point of indifference is reached between health state A and a health state B. At this point of indifference, it is then possible to calculate the corresponding utility value for that health state B, which represents an individual’s health preference for that state.

For example, suppose an individual is indifferent between living for six years in health state A (full health) and ten years in a health state B denoted as health state (32242). In that case, the utility valuation is calculated for that health state B as *x/t = 6/10 = 0.6.* This format is known as ‘Better Than Dead’ (BTD), resulting in a positive utility valuation.

**Calculating a health state utility value for a worse than dead state**

An individual can also place a ‘Worse Than Dead’ (WTD) valuation on a health state B if they decide that health state B is so severe that they would rather die than be indifferent between any allotted time in health state A and ten years in that health state B. If so, the question continues in a different format with the same health state B. Again, the amount of time in health state A (full health) is altered on a scale of one to ten years while the amount of time in health state B is fixed now at 20 years in the WTD format. The iterative procedure resumes until the individual is indifferent between the two lives. It is then possible to calculate the utility value for that health state B in the WTD format, resulting in a negative utility valuation. More detail on cTTO health states and how the utility values are derived can be found in supplementary material and in the paper by Oppe et al. (2016)

For example, suppose an individual is indifferent between living for two years in health state A (full health) and 20 years in that health state B denoted as health state (45455) in the worse than dead (WTD) format. In that case, the utility valuation is calculated for that health state B as *(x – t)/t = (2 – 10)/10 = -0.8*. It is possible to place a utility valuation of zero on a health state B entirely resulting in a utility valuation of -1.

**Table 1A:** Random effects tobit model

|  |  |  |
| --- | --- | --- |
|  | **cTTO disutility** | |
| **Variables** | **Coefficient** | **Standard Error** |
| **Mobility**  Slight problems | 0.081*** | 0.028 |
| Moderate problems | 0.081*** | 0.029 |
| Severe problems | 0.154*** | 0.029 |
| Unable | 0.210*** | 0.029 |
| **Self-care**  Slight problems | 0.035 | 0.029 |
| Moderate problems | 0.116*** | 0.030 |
| Severe problems | 0.311*** | 0.032 |
| Unable | 0.272*** | 0.028 |
| **Usual activities**  Slight problems | 0.010 | 0.027 |
| Moderate problems | 0.027 | 0.028 |
| Severe problems | 0.160*** | 0.029 |
| Unable | 0.142*** | 0.027 |
| **Pain/discomfort**  Slight | 0.043 | 0.028 |
| Moderate | 0.144*** | 0.029 |
| Severe | 0.440*** | 0.029 |
| Extreme | 0.588*** | 0.027 |
| **Anxiety/depression**  Slight | 0.066*** | 0.027 |
| Moderate | 0.143*** | 0.029 |
| Severe | 0.410*** | 0.029 |
| Extreme | 0.538*** | 0.028 |
| Age | 0.005* | 0.003 |
| Gender | 0.026 | 0.061 |
| Married | -0.060 | 0.072 |
| Urban household | -0.030 | 0.060 |
| Constant | -0.113 |  |
| Number of observations | 242 |  |

***p< 0.01 **p<0.05 *p<0.1

Dependent variable = cTTO disutility value; base/reference category = no problems in each health dimension.

A random effects Tobit model is used to account for censoring, and to account for multiple observations per respondent.

The 20 main effects are included only as a means to control for the health state valued by each respondent. As the health dimension increase in severity the disutility value increases as we would expect. Some illogical inconsistencies exist for the Self-care and Usual activities dimensions where the severe problems in both dimension has a greater disutility value than the extreme problems category. This may be attributed to random error, and it is typically not considered any reason for concern as noted by Versteegh et al. (2016). The logical inconsistencies may be attributable to our small sample size. The other socio-demographic variables included have no bearing on health preferences, but this equally could be in part due to our small sample size again. The variables were included as they are shown to be significant predictors of health state utility valuations, as noted by Sayah et al. (2016) who used a much larger sample size in their analysis. This point is also noted in the paper.

M Versteegh, M., M Vermeulen, K., M A A Evers, S., de Wit, G.A., Prenger, R., A Stolk, E., 2016. Dutch tariff for the five-level version of EQ-5D. Value Health 19, 343–352.

**Table 2A:** Bivariate probit average marginal effects for GP use

|  | 1. **(GP) Cancer screening** | | 1. **(GP) Blood pressure** | | 1. **(GP) Cholesterol test** | | 1. **(GP) Blood test** | | 1. **(GP) Urine test** | |
| --- | --- | --- | --- | --- | --- | --- | --- | --- | --- | --- |
| Variable | Coefficient | (SE) | Coefficient | (SE) | Coefficient | (SE) | Coefficient | (SE) | Coefficient | (SE) |
| Health preference | 0.472 | 0.506 | 0.549 | 0.516 | 0.510 | 0.509 | 0.562 | 0.512 | 0.492 | 0.511 |
| Third level education | -0.182*** | 0.040 | -0.176*** | 0.041 | -0.179*** | 0.041 | -0.179*** | 0.041 | -0.175*** | 0.041 |
| Sex | -0.135*** | 0.053 | -0.134*** | 0.054 | -0.134*** | 0.053 | -0.134*** | 0.054 | -0.132*** | 0.053 |
| §Health coverage | (base: no coverage) |  |  |  |  |  |  |  |  |  |
| §Medical card | 0.191*** | 0.048 | 0.192*** | 0.050 | 0.198*** | 0.047 | 0.195*** | 0.047 | 0.190*** | 0.048 |
| §Private insurance | -0.020 | 0.056 | -0.008 | 0.057 | -0.015 | 0.057 | -0.019 | 0.057 | -0.018 | 0.056 |
| VAS | -0.004* | 0.002 | -0.004 | 0.002 | -0.004 | 0.002 | -0.004* | 0.002 | -0.004 | 0.002 |
| Employed | 0.017 | 0.050 | 0.016 | 0.051 | 0.019 | 0.051 | 0.018 | 0.051 | 0.022 | 0.051 |
| Age | 0.241*** | 0.078 | 0.232*** | 0.081 | 0.238*** | 0.079 | 0.236*** | 0.078 | 0.228*** | 0.079 |
| Married | 0.214*** | 0.063 | 0.198*** | 0.066 | 0.209*** | 0.064 | 0.207*** | 0.064 | 0.206*** | 0.065 |
| Number of observations | 242 |  | 242 |  | 242 |  | 242 |  | 242 |  |

***p< 0.01 **p<0.05 *p<0.1

Dependent variable = whether or not a respondent had a GP visit.

SE = standard error.

§base/reference category = no health coverage.

**Table 3A:** Variation in (55555) utility valuation by socio-demographic characteristics

| **Variable** | **Observations** | **Mean** | **Standard error** | **T-stat** |
| --- | --- | --- | --- | --- |
| Age $\leq$ 36 | 132 | 1.77 | 0.03 | 11.02 |
| Age > 36 | 110 | 1.84 | 0.07 |  |
| Male | 94 | 1.82 | 0.06 | -4.60 |
| Female | 148 | 1.78 | 0.05 |  |
| Married | 115 | 1.81 | 0.07 | -1.92 |
| Not married | 127 | 1.8 | 0.05 |  |
| Urban | 143 | 1.78 | 0.05 | 7.39 |
| Rural | 99 | 1.83 | 0.06 |  |

T-stat is testing the statistical difference between categories in each variable.

There is a statistical difference in the predicted utility valuation of the (55555) health state across the categories of socio-demographic variables included.

**Table 4A:** Variation in (55555) utility valuation by each preventive service

| **Variable** | **Observations** | **Mean** | **Standard error** | **T-stat** |
| --- | --- | --- | --- | --- |
| Cancer screening (Yes) | 102 | 1.80 | 0.07 | -1.00 |
| Cancer screening (No) | 140 | 1.79 | 0.05 |  |
| Blood pressure (Yes) | 163 | 1.80 | 0.06 | -2.48 |
| Blood pressure (No) | 79 | 1.78 | 0.05 |  |
| Cholesterol (Yes) | 112 | 1.81 | 0.06 | -4.15 |
| Cholesterol (No) | 130 | 1.78 | 0.05 |  |
| Blood test (Yes) | 150 | 1.80 | 0.06 | -1.40 |
| Blood test (No) | 92 | 1.79 | 0.05 |  |
| Urine test (Yes) | 78 | 1.80 | 0.07 | -0.51 |
| Urine test (No) | 164 | 1.80 | 0.06 |  |
| GP (Yes) | 194 | 1.79 | 0.06 | -1.6 |
| GP (No) | 48 | 1.80 | 0.06 |  |

T-stat is testing the statistical difference between categories in each variable.

We examined the variation in the predicted utility valuation of the (55555) health state across the categories of each preventive service (i.e. used the service or not). The results show significant differences exist between those who have and have not had a blood pressure check and those who have and have not had cholesterol check.

**Table 5A:** Counts of those who availed of each preventive service and did or did not attend the GP in the past year.

| **Variable** | **No GP visit** | **Yes GP visit** | **Total** |
| --- | --- | --- | --- |
| Cancer Screening | 11 | 91 | 102 |
| Blood pressure check | 19 | 144 | 163 |
| Cholesterol | 12 | 100 | 112 |
| Blood test | 15 | 135 | 150 |
| Urine test | 5 | 73 | 78 |

This sub analysis looks at the counts of those who have availed of each preventive service and whether they had a GP visit in the past year.

As can be seen from the above results, the majority of individuals who availed of preventive care also had a GP visit in the past year. This is to be expected, as GPs can carry out a range of preventive tests and can also refer patients on for further diagnostic tests.

**Table 6A:** Total number of tests

| **Number of tests** | **Number of respondents** | **Percent %** |
| --- | --- | --- |
| 0 | 42 | 17.36 |
| 1 | 32 | 13.22 |
| 2 | 42 | 17.36 |
| 3 | 47 | 19.42 |
| 4 | 47 | 19.42 |
| 5 | 32 | 13.22 |
| **Total** | **242** | **100** |

Numerous individuals had multiple tests. This is to be expected considering a GP or other healthcare provider could potentially carry out all of the preventive tests in one visit.

**Table 7A:** Variation in VAS by Age

| **Variable** | **Observations** | **Mean** | **Standard error** | **T-stat** |
| --- | --- | --- | --- | --- |
| Age $\leq$ 36 | 132 | 85 | 10.19 | -1.62 |
| Age > 36 | 110 | 83 | 11.83 |  |

T-stat is testing the statistical difference between categories in the age variable.

There is no statistical difference between the age categories regarding VAS.
